# The Effect of Thermal Contact Number on the Tube–Tube Contact Conductance of Single-Walled Carbon Nanotubes

**DOI:** 10.3390/nano9030477

**Published:** 2019-03-23

**Authors:** Xueming Yang, Xinyao Zhang, Bingyang Cao

**Affiliations:** 1 Department of Power Engineering, North China Electric Power University, Baoding 071003, China; zxy_ncepu@sina.com; 2 Key Laboratory for Thermal Science and Power Engineering of Ministry of Education, Department of Engineering Mechanics, Tsinghua University, Beijing 100084, China; caoby@tsinghua.edu.cn

**Keywords:** carbon nanotubes, contact conductance, molecular dynamics simulations

## Abstract

The contact conductance of single, double, and triple thermal contacts of single-walled carbon nanotubes (SWCNTs) was investigated using molecular dynamics simulations. Our results showed that the effect of the thermal contact number on the contact conductance was not as strong as previously reported. The percentages of contact conductance of double and triple thermal contacts were about 72% and 67%, respectively, compared to that of a single thermal contact. Moreover, we found that the contact conductance of the double and triple thermal contacts was associated with the SWCNT length and the positional relationship of the thermal contacts.

## 1. Introduction

Individual carbon nanotubes (CNTs) [1] have extraordinarily high thermal conductivity. The theoretical study by Berber et al. [2] showed that the thermal conductivity of individual CNTs reached 6000 Wm^−1^K^−1^ at room temperature. The thermal conductivity of individual single-walled CNTs (SWCNTs) measured by Pop et al. [3] was close to 3500 Wm^−1^K^−1^ at room temperature. Therefore, CNTs have broad application prospects in the field of thermal management due to their excellent thermal conductivity. They can be used to improve the thermal conductivity of polymer matrices and for the construction of self-supporting CNT network structures. The thermal conductivity of a CNT network can be tuned by changing the orientation, distribution, and connection of individual CNTs.

In terms of microstructure, macroscopic CNT materials are generally composed of numerous CNTs that are overlapped or connected to form a spatial network structure. A random network structure is the most common microstructure observed in macroscopic CNT materials. The thermal conductivity of macroscopic CNT materials is closely related to the microstructural parameters of the CNTs, including the length, tube diameter, chirality, arrangement, and orientation. Moreover, it is limited by the CNT vacancies, defects, and external chemical adsorption or chemical modification, because the chemical adsorption and irregular internal structures reduce the phonon mean free path of the CNT materials and thus decrease the thermal conductivity [4,5,6,7,8].

In addition, studies have thus far indicated that one of the main reasons for the limited thermal conductivity of randomly oriented CNT packed beds and CNT composite materials is the weak thermal coupling [9,10,11,12,13,14,15,16,17] among the CNTs. For randomly oriented CNT-based macroscopic systems, the weak van der Waals interaction among the CNTs limits the overall performance of the material; thus, the large tube–tube thermal contact resistance is an important reason for the limited thermal conductivity. Unfortunately, simulations and theoretical analyses are still quite difficult to perform because the random network structure of macroscopic CNT materials is extremely complex.

There are very few theoretical prediction models for the thermal conductivity of a random CNT network, such as the models for a non-welded random network proposed by Chalopin et al. [14,15] and Volkov et al. [16,17] and the model for a partially welded random network proposed by the authors [18]. These models have been used in the thermal conductivity analysis of experimentally fabricated SWCNT films or packed beds [9,19,20]. In these models, the tube–tube contact conductance of the thermal contacts is one of the key parameters. Prasher et al. [9] found that the tube–tube contact conductance is related to the number of thermal contacts. When a single CNT and two CNTs constitute two thermal contacts, the tube–tube contact conductance is nearly 1/10 that of a single thermal contact. So far, the contact conductance of multiple thermal contacts of CNTs has not yet been investigated; however, research on this topic is necessary because multiple thermal contacts are common in random CNT networks. Understanding and clarifying the tube–tube contact conductance of multiple thermal contacts is important for the prediction of the thermal conductivity of randomly oriented CNT networks.

## 2. Materials and Methods

**Figure 1:**
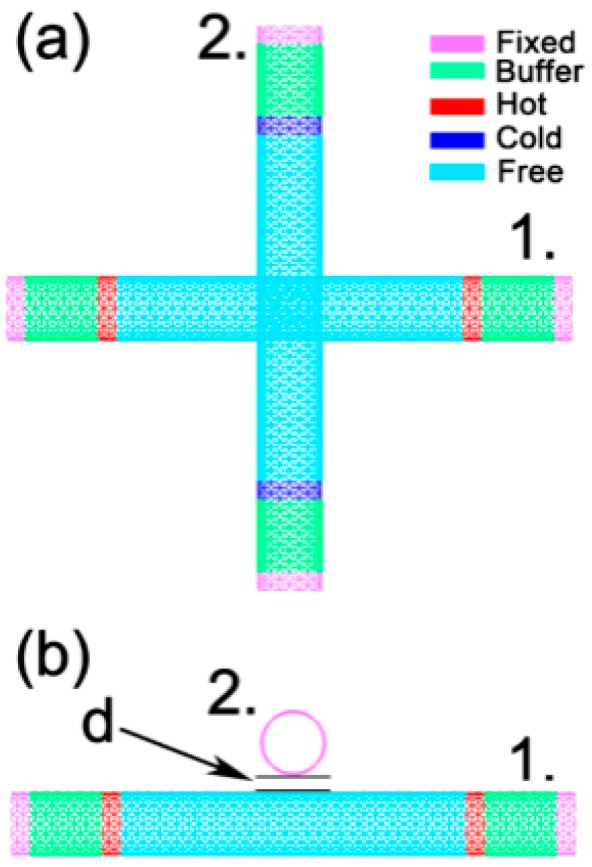
The calculation model for the thermal contact conductance of two carbon nanotubes with a single thermal contact: (**a**) aerial view; (**b**) front view.

The general model for calculating the tube–tube contact conductance of a single thermal contact is shown in Figure 1. To facilitate comparisons with the previous study by Prasher et al. [9], in the molecular dynamics (MD) simulations, the selected SWCNTs are all CNTs (10, 10), the crossing angle of the pair of tubes is 90°, and the wall-to-wall distance (d) between the two tubes is the equilibrium distance of 3.4 Å [9]. In the MD calculations, the tube–tube contact conductance does not depend on whether the SWCNTs are semiconducting or metallic CNTs, because only the contribution from phonons in the adjacent CNTs is considered and the surface mass density for both semiconducting CNTs and metallic CNTs are the same. The individual CNT length is set at 50 unit cells, and each unit cell length is 2.45951 Å with 40 atoms per unit cell. Free boundary conditions are used in all three directions. Both ends of the CNTs are fixed to prevent translation or rotation during the simulations, the fixed length is 2 unit cells, and the fixed part is marked in purple, as shown in Figure 1. The lengths of the two hot slabs of tube 1 are 2 unit cells and are marked in red. The lengths of the two cold slabs of tube 2 are also 2 unit cells and are marked in dark blue. The wall-to-wall distance between tube 1 and tube 2 is *d*. The buffer regions are 6 unit cell lengths between the fixed slabs and hot (cold) slabs which act as thermal shields against thermal reflection at the tube’s end [21,22,23].

The LAMMPS package [24] and the adaptive intermolecular reactive empirical bond order (AIREBO) potential [25] are used in the MD simulations. The AIREBO potential is composed of three parts:

(1) $$E=\frac{1}{2}\sum_{i}\sum_{j\neq i}\left[E_{ij}^{REBO}+E_{ij}^{LJ}+\sum_{k\neq i,j}\sum_{l\neq i,j,k}E_{ijkl}^{TORSION}\right]$$

where $E_{ij}^{REBO}$ is the term of the REBO potential; $E_{ij}^{LJ}$ is similar to the long-distance interaction term of the standard Leonard–Jones potential; $E_{ijkl}^{TORSION}$ is the four-body potential torsion term that depends on the dihedral angle. This potential has been widely used to investigate the thermal transport properties of CNT- or graphene-based nanostructures [26,27,28]. In the molecular simulations in this study, the cutoff distance $E_{ij}^{LJ}$ is 1.02 nm. The term $E_{ijkl}^{TORSION}$ is closed due to the small contribution to thermal transport compared to the first two terms, and the open mode of this term will significantly increase simulation time. This treatment is consistent with that used in many previous studies [27,28].

As the applied temperature gradient method convergence rate is relatively slow, the applied heat flux method [29,30,31] is used to simulate the thermal transport. In this method, constant kinetic energy is provided to the hot slabs and the same kinetic energy is removed from the cold slabs during a fixed time interval to create a temperature gradient. The atomic velocity changes in the hot slabs and cold slabs are controlled by the scaling factor *R* and variable $v_{sub}$. The atomic velocity is defined as follows:

(2) $${v_{i}}^{\prime }=Rv_{i}-v_{sub}\;i\in N$$

where ${v_{i}}^{\prime }$ and $v_{i}$ represent the new and old velocities of atom *i* before and after certain time steps and N represents the set of atoms in the control area. The expressions of *R* and $v_{sub}$ are as follows:

(3) $$R=\sqrt{\frac{E_{k}^{\prime }-\frac{1}{2}\frac{P^{2}}{M}}{E_{k}-\frac{1}{2}\frac{P^{2}}{M}}}=\sqrt{\frac{E_{k}+\Delta E_{k}-\frac{1}{2}\frac{P^{2}}{M}}{E_{k}-\frac{1}{2}\frac{P^{2}}{M}}}$$

(4) $$v_{sub}=\frac{(R-1)P}{M}=\frac{(R-1)\sum_{i\in N}m_{i}v_{i}}{\sum_{i\in N}m_{i}}$$

where $E_{k}$ is the new total kinetic energy of sub-region *N*; $E_{K}^{\prime }$ is the old total kinetic energy of sub-region *N*; $\Delta E_{K}$ is the difference between the old and new kinetic energies; *P* is the total momentum of sub-region *N*; *M* is the total atomic mass of sub-region *N*.

In each non-equilibrium MD (NEMD) simulation for calculating the tube–tube contact thermal conductance, a relaxation of the system is first performed in an NVT ensemble at 300 K for 1.2 × 10^6^ time steps (0.5 fs/time step) using the Nosé–Hoover thermostat. Then, to establish a stable temperature difference between the tubes, an NEMD simulation is conducted for another 6 × 10^6^ steps in the NVE ensemble, in which a constant amount of kinetic energy is added/subtracted to/from the hot/cold slabs at a regular interval (10 time steps). The output of the last 1 × 10^6^ steps is used to calculate the temperature profile of the tubes.

We use some simplified terminology in this paper for the sake of convenience. A single thermal contact refers to a CNT and another CNT that are closely staggered in space with heat conduction occurring between the two CNTs. Double thermal contacts and multiple thermal contacts refer to a CNT and two (or more) CNTs that are staggered in space with heat conduction occurring among the CNTs. The tube–tube contact conductance of double and multiple thermal contacts refers to the contact conductance of every single thermal contact among the multiple thermal contacts, rather than the overall contact conductance. For a symmetrical structure, the average value can be taken as the tube–tube contact conductance.

## 3. Results and Discussion

### Contact Conductance

**Figure 2:**
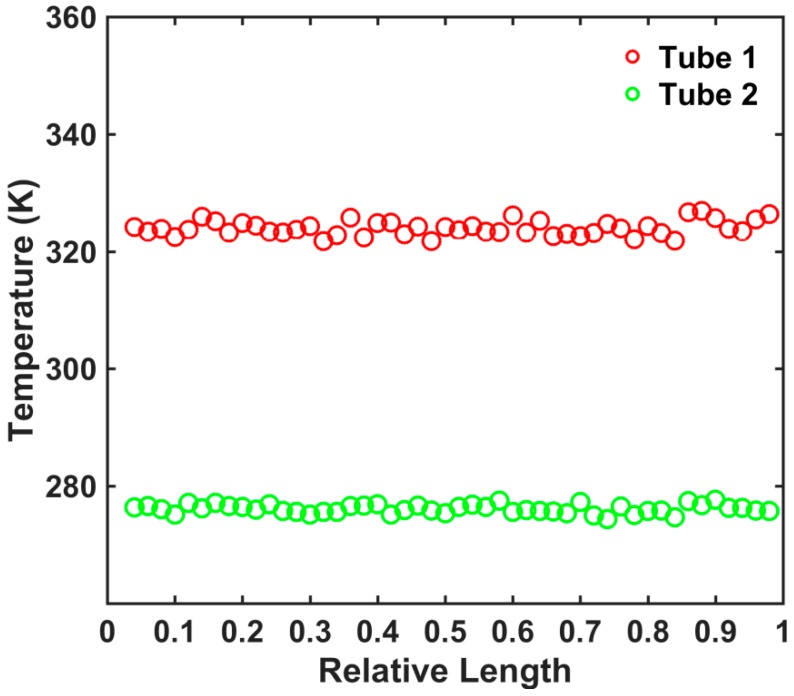
Typical temperature profile of the two single-walled carbon nanotubes (SWCNTs) in a single thermal contact.

The model for calculating the tube–tube contact conductance of a single thermal contact is shown in Figure 1. The amounts of heat flow into each hot slab and out of each cold slab are both expressed as $Q_{0}$ in units time. The $Q_{0}$ selection is not a fixed value in different models and simulation systems. The temperature difference between the tubes is maintained in a suitable range by estimating and selecting the appropriate $Q_{0}$ value. The typical temperature profiles of the two CNTs in the single thermal contact are shown in Figure 2.

In our previous work [18,23], we investigated the inter-tube thermal conductance of two CNTs connected by a molecular junction. The temperature profile of these two CNTs exhibited a temperature gradient, namely, the temperature difference between the two tubes was small near the molecular junction and increased gradually with increasing distance from the molecular junction. In the calculation model in this study, the temperature is basically the same for the hot slabs and cold slabs and changes little. The tube–tube contact conductance is defined as $\sigma_{c}=Q/\bar{\Delta T_{C}}$, where $\bar{\Delta T_{c}}$ is the average temperature of the two tubes in the direction of the axial length and *Q* is the transitive energy through the thermal contact between the two tubes per unit time.

When the *d* between the two tubes is 3.4 Å, the calculated tube–tube contact conductance $\sigma_{c}$ = 40.3 pW/K is very similar to the result of 43.1 pW/K reported by Evans et al. [27], who used the Tersoff and Lennard–Jones (LJ) potential in an MD simulation. The value is also close to the tube–tube contact conductance of 50 pW/K obtained by Prasher et al. [9], who used the MD method based on the Tersoff and LJ potential and the atomic Green’s function. Since there is no chemical bond connection between the CNTs, the atomic interaction between the CNTs is well described by the LJ potential.

In a previous study by Prasher et al. [9], a single CNT and two parallel CNTs constituted two thermal contacts, the distance between the two thermal contacts was 8.16 Å, and the wall-to-wall distance of the two CNTs which comprised each thermal contact was 3.4 Å; their results showed that the tube–tube contact conductance was less than 1/10 of that of the single thermal contact. No explanation was provided for this mechanism, and the authors only suggested that the tube–tube contact conductance of multiple thermal contacts required further study. Since tube–tube contact conductance is one of the basic parameters required for the calculation and analysis of the thermal conductivity of CNT random networks, it is necessary to understand whether and how the thermal contact number affects the tube–tube contact conductance of single-walled CNTs.

**Figure 3:**
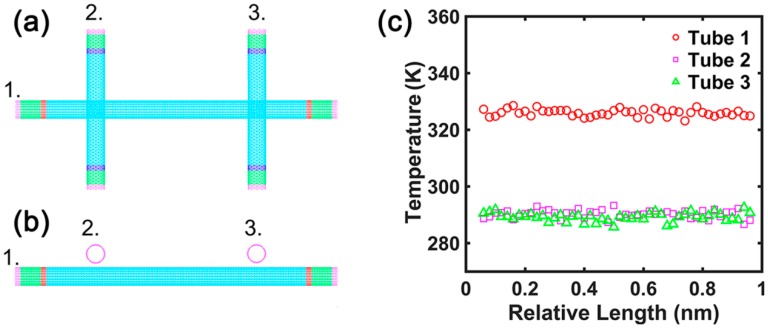
The calculation model 1 for the contact conductance of the double thermal contacts: (**a**) aerial view; (**b**) front view. (**c**) Typical temperature profile of the three carbon nanotubes in the double thermal contacts.

Firstly, we selected the calculation model 1 shown in Figure 3a,b. The distance (*d*) between the walls of tube 1 and tube 2 or tube 3 is 3.4 Å. The distance (L) between tube 2 and tube 3 is 50 unit cells. The length of CNT 1 is 100 unit cells, while the lengths of CNT 2 and CNT 3 are both 50 unit cells. In the thermal contacts obtained by simulation, the temperature profile of the three CNTs along the direction of the tube lengths is shown in Figure 3c. The calculated tube–tube contact conductance is 40.15 pW/K, which is close to the tube–tube contact conductance of the single thermal contact.

**Figure 4:**
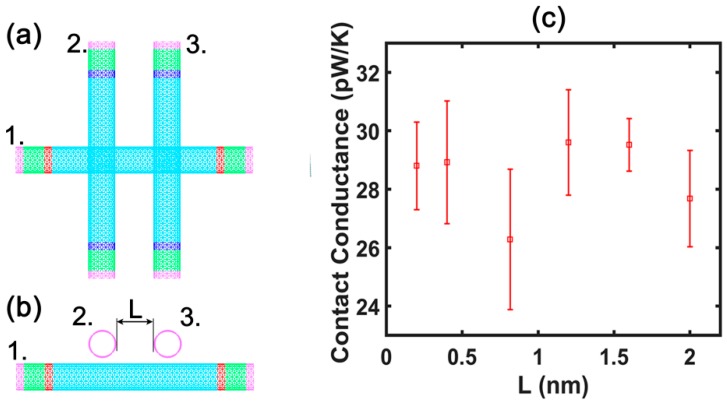
The calculation model 2 for the contact conductance of the double thermal contacts: (**a**) aerial view; (**b**) front view. (**c**) The variation of the contact conductance of the double thermal contacts with the distance between tubes 2 and 3.

Moreover, to investigate whether the distance between tube 2 and tube 3 affects the contact conductance of double thermal contacts, calculation model 2 is used as shown in Figure 4a,b. We selected distances of 2 Å, 4 Å, 8.16 Å, 12 Å, 16 Å, and 20 Å, respectively, between tube 2 and tube 3. The lengths of tubes 1–3 in the setup are 50 unit cells. The results of the contact conductance calculation, as shown in Figure 4c, suggest that the distance between tube 2 and tube 3 does not significantly affect the value of the contact conductance. However, a comparison with the calculation results of tube 1 (Figure 3) with 100 unit cells indicates that the tube length exerts a significant effect on the contact conductance; the longer the tube length, the larger the contact conductance. This is consistent with the relationship between contact conductance and tube length discovered by Hu et al. [32] in their analysis of tube–tube contact conductance of a single thermal contact.

**Figure 5:**
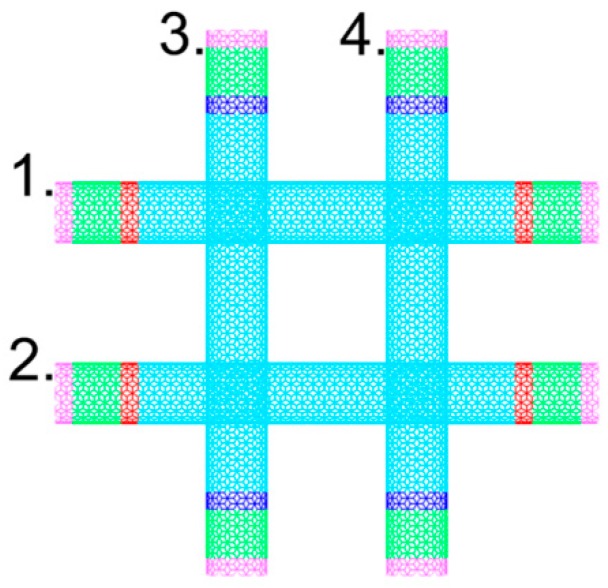
The calculation model 3 for the contact conductance of the double thermal contacts.

In calculation model 2, the energy per unit time applied to each hot slab in tube 1 is 2*Q*_0_, while that removed from each cold slab in tube 2 and tube 3 per unit time is *Q*_0_. In the calculation, we assumed that the amount of heat conducted from tube 1 to tube 2 or tube 3 per unit time is *Q*_0_. However, it still has to be validated whether the tube–tube contact conductance can be calculated directly by using the average temperature between tube 1 and tube 2 or tube 1 and tube 3. We developed a symmetric calculation model 3 for this purpose consisting of four carbon nanotubes, as shown in Figure 5. The lengths of the CNTs in this setup are 50 unit cells and the positional relationship of each thermal contact is symmetrical. The amounts of heat flow into the hot slabs and out of the cold slabs are both *Q*_0_. The average contact conductance of the thermal contacts calculated by this model is 28.95 pW/K, which is close to that of the double thermal contacts obtained via model 2. That is to say, our calculation method is suitable and effective. These simulation results indicate that the phenomenon reported by Prasher et al. [9], i.e., that the tube–tube contact conductance of the double thermal contacts is 1/10 that of the single thermal contact, was not observed in our simulations.

**Figure 6:**
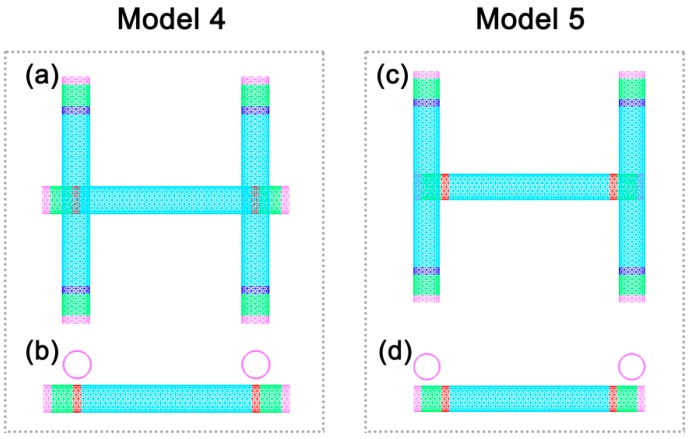
Calculation model 4 and model 5 for the contact conductance of the double thermal contacts: (**a**,**c**) aerial view; (**b**,**d**) front view.

Although the calculation model by Prasher et al. is similar to our calculation model 2, the tube 2 and tube 3 were placed at both ends of the original tube in their setup. Therefore, the question arises whether the results by Prasher et al. were caused by the specific positional relationship of tube 2 and tube 3 to tube 1. To test this hypothesis, we conducted further simulations. Calculation models 4 and 5 (Figure 6) were developed to represent two scenarios in which tube 2 and tube 3 are placed near both ends of tube 1. The contact conductance is 17.42 pW/K for calculation model 4 and 13.33 pW/K for calculation model 5.

When each thermal contact of the double thermal contacts is placed at both ends of tube 1, the contact conductance is reduced to nearly half that of calculation model 2. Therefore, in calculation model 2, the relative positions of tubes 2 and 3 along the axial direction of tube 1 and the distance from the ends affect the contact conductance. However, the change is minimal compared to the nearly 10-fold difference observed previously [9]. When each thermal contact of the double thermal contacts is placed at both ends of tube 1, the heat transfer area of the surface of the adjacent CNTs is reduced to nearly half the original value. This results in a reduction in contact conductance of about half compared to calculation model 2.

**Figure 7:**
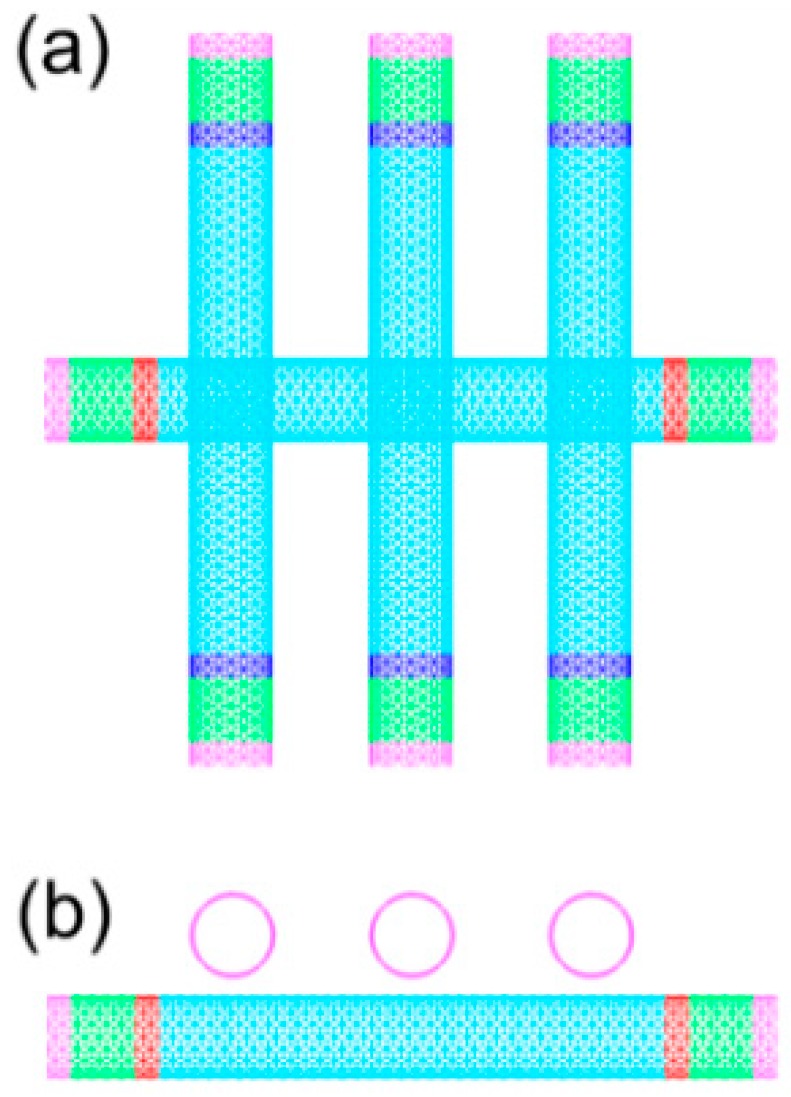
Calculation model for the contact conductance of the three thermal contacts: (**a**) aerial view; (**b**) front view.

We constructed a calculation model for the case of triple thermal contacts, namely, an individual CNT and three CNTs which constituted three thermal contacts, as shown in Figure 7. The average tube–tube contact conductance of the three thermal contacts obtained by calculation was 26.86 pW/K, which was slightly smaller than the contact conductance of the double thermal contacts obtained by calculation model 2.

The simulation results indicate that the contact conductance decreases with the increase in the number of thermal contacts. It should be noted that, to date, we have not evaluated the results obtained by Prasher et al. due to a lack of experimental data. However, one of the objectives of this study was to describe the discrepancies. Although it is reasonable to assume that the lower value of the contact conductance may be attributable to the thermal coupling of the multiple thermal contacts, a quantitative explanation of the mechanism is still quite challenging. Further experimental study of the effect of the thermal contact number on the tube–tube contact conductance is strongly recommended.

## 4. Conclusions

In this study, we used MD simulations to study the tube–tube contact conductance of single-walled CNTs. Several calculation models were employed to analyze the tube–tube contact conductance of single, double, and triple thermal contacts under different circumstances. Our results showed that the effect of the thermal contact number on the contact conductance was not as strong as that reported by Prasher et al. The contact conductance of the double and triple thermal contacts were about 72% and 67%, respectively, compared to that of a single thermal contact. Moreover, we found that the contact conductance of the double and triple thermal contacts was also associated with the CNT length and the positional relationship of the thermal contacts.

In conclusion, our work offers new insights into the effect of thermal contact number on the tube–tube contact conductance of SWCNTs. The tube–tube contact conductance is crucial for the understanding of the overall thermal conductivity of CNT-based macroscopic systems. We have to acknowledge that our study is limited and only focused on a finite-sized SWCNT system with double and triple thermal contacts. Further theoretical and experimental studies for more generalized SWCNTs systems with multiple thermal contacts are still needed.
